# Emergence and Characterization of Three Pseudorabies Variants with Moderate Pathogenicity in Growing Pigs

**DOI:** 10.3390/microorganisms13040851

**Published:** 2025-04-09

**Authors:** Zhendong Zhang, Cong Wang, Chengyue Wu, Qingteng Wei, Zhengqin Ye, Wenqiang Wang, Zhe Sun, Kegong Tian, Xiangdong Li

**Affiliations:** 1 Jiangsu Co-Innovation Center for Prevention and Control of Important Animal Infectious Diseases and Zoonoses, College of Veterinary Medicine, Yangzhou University, Yangzhou 225009, China; 008686@yzu.edu.cn (Z.Z.); mx120220948@stu.yzu.edu.cn (Q.W.); yezhengqin@126.com (Z.Y.);; 2 China Animal Husbandry Industry Co., Ltd., Beijing 100070, China; 3 College of Veterinary Medicine, Nanjing Agricultural University, Nanjing 210095, China; 4 National Research Center for Veterinary Medicine, Luoyang 471003, China

**Keywords:** pseudorabies virus, complete genome, genetic characterization, evolution, pathogenicity

## Abstract

Pseudorabies virus (PRV) remains a critical threat for the global swine industry, with heightened attention due to the emergence of variant strains since late 2011 in China. Emergent viral variants generally undergo three to four years of adaptation to present new phenotypes. However, limited investigations have been performed on the evolution and pathogenicity of variant PRV strains in growing pigs after 2015. In this study, three PRV field strains, named SD1501, SD1701, and SD1801, were isolated and their genetic characteristics and pathogenicity on 9-week-old pigs were analyzed. Nucleotide identity and phylogenetic analyses based on the complete genome sequence, as well as major immunogenic and virulence-related genes revealed that all three isolates clustered closely with genotype II variant strains prevalent in China. The pathogenicity analysis demonstrated that the three isolates exhibited moderate pathogenicity in growing pigs with a TCID_50_ of 10^7^. Infected pigs displayed transient fever and reduced appetite, with only one pig in each challenge group showing typical neurological symptoms and succumbing within 6 days post infection. These findings enrich the epidemiological data of PRV and provide direct evidence for the phenotypic variations caused by PRV infection, which enhances our understanding of PRV evolution in China and contributes to PRV control in the field.

## 1. Introduction

In 1813, a disease manifesting with a “mad itch” broke out in bovines in the United States, and was identified as pseudorabies virus (PRV) infection about one hundred years later [1,2]. PRV, also known as Aujeszky’s disease virus (ADV) or Varicellovirus suidalpha1, belongs to the family *Orthoherpesviridae*, subfamily *Alphaherpesvirinae,* genus *Varicellovirus* (https://ictv.global/taxonomy, accessed on 15 August 2024). PRV has been detected in a wide range of animals, but pigs are the natural host and reservoir [3,4]. Since the 1970s, with the global development of the swine industry, outbreaks of PR cases have occurred worldwide, causing significant economic losses [5,6]. Although PRV has been eliminated from domestic pigs in many countries, it is still popular in most pig-producing countries (https://www.woah.org/en/home/) and it still also persists in wild boars [7]. Similarly to other alphaherpesviruses, PRV can persist in the peripheral nervous system and establish latent infection [8]. The irregular reactivation of PRV by stress or other factors complicates prevention and control efforts in the field [9].

PRV infection leads to abortion and stillbirth for pregnant sows, while in growing pigs, clinical symptoms are characterized by central nervous system (CNS) disorders, growth retardation, and weight loss [10]. The infection is often fatal in piglets less than two weeks old [10]. Due to the similar clinical presentation of PR and several common swine diseases, such as porcine reproductive and respiratory syndrome (PRRS) and porcine circovirus 2 (PCV2), gE antibody analysis, qPCR, and sequencing methods are used to preform differential diagnosis. The first complete PRV genome sequence was obtained in 2004 using fragments derived from six distinct strains [11]. With the advent and application of high-throughput sequencing technologies, an increasing number of PRV genomes have been sequenced and characterized. The PRV genome is about 145 kb in length and encodes over 70 proteins with structural, virulence, and regulatory roles. Among the PRV genes, the glycoproteins gB, gC, gD, and gE and thymidine kinase (TK) are the most significant virulence-related genes [3,12,13]. As one of the most variable genes, the gC gene is commonly and widely used for genotyping and divides PRV strains into two genotypes, I and II [14]. Genotype I predominantly includes European and American PRV isolates, while Genotype II strains are mostly prevalent in China and other Asian countries [6,8,14].

In China, the evolution of PRV follows two different stages, corresponding to classical and variant strains (both within Genotype II) [15]. Prior to 2011, PRV outbreaks caused by classical strains were effectively controlled in most pig farms in China, owing to the sufficient protection of Bartha-K61 vaccine. However, variant PRV strains have been identified in pigs vaccinated with Bartha-K61 since late 2011, which exhibited unique amino acid insertions, deletions, and mutations in the key protective proteins [16,17]. These variant strains also displayed faster transmission, increased pathogenicity, and more severe clinical manifestations compared to classical strains [18,19]. In response, modified vaccines, adjusted vaccination schedules, improved biosecurity protocols, and elimination strategies have been implemented. Despite considerable progress in PRV control, evidenced by declining gE antibody levels and reduced PCR positivity rates [6], it still remains crucial to continuously monitor and understand the evolution of PRV strains in China [20].

In general, emergent viral variants typically undergo three to four years of adaptation before experiencing diminished prevalence, potentially due to host immune recognition and competitive exchange by evolved novel strains. However, scarce reports exist on the complete genome and pathogenicity analyses of PRV isolates from 2015 to 2018. In the present study, three variant PRV strains, namely, SD1501, SD1701, and SD1801, were successfully isolated in 2015, 2017, and 2018, respectively. Moreover, we analyzed the complete genome sequence, evolutionary dynamics, variations, and pathogenicity of the three isolates in growing pigs.

## 2. Materials and Methods

### 2.1. Virus Isolation and Identification

Vero cells were cultured in Dulbecco’s modified Eagle medium (DMEM) (Solarbio, Shanghai, China), supplemented with 10% fetal bovine serum (FBS, NULEN BIOTECH, Shanghai, China), and maintained at 37 °C in 5% CO_2_. Three sow farms with a vaccination history with the PRV Bartha-K61 vaccine (three times/year/sow) in Shandong Province of China experienced severe cases evidenced by abortion in sows and neurological symptoms in suckling piglets in 2015, 2017, and 2018, which were confirmed to be PRV variants through PCR detection and gE sequencing, without other pathogens (PRRS, PCV2, etc.). The reserved PRV-positive samples (homogenate of mixed tissues) were filtered through a 0.22 μm filter and inoculated into Vero cells for virus isolation. Vero cells cultured in 6-well plates were infected with 500 μL of the filtered supernatant for 1.5 h; then, the cells were washed with phosphate-buffered saline (PBS) and incubated in DMEM supplemented with 2% FBS. Infected cells were harvested until the presence of approximately 80% cytopathic effects (CPEs). A plaque assay was performed to purify the isolated virus. After 24 h post infection (hpi) in 6-well plates, the viral solution was removed, and agarose solution was added by mixing 1:1 agarose solution with 2× DMEM nutrient medium supplemented with double antibiotics and 1% serum. The plate was placed at 4 °C for 3 min and incubated at 37 °C for 2–3 days. When visible plaques appear, the medium was removed, and the cells were fixed with 4% paraformaldehyde for 10 min. Following a wash with PBS and staining with 200 μL crystal violet dye, the plaque was observed.

To confirm the isolated virus, an indirect immunofluorescent assay (IFA) was performed: Vero cells were infected with the isolates for 24 h, washed three times with PBS, and permeabilized with pre-cooled absolute ethanol for 30 min at room temperature. After removing the ethanol and washing with PBS, the cells were incubated overnight at 4 °C with PRV gB protein antibody (1:1000) and IE180 protein antibody (1:500), kindly gifted by Prof. Beibei Chu at Henan Agricultural University. Following three PBS washes, the cells were then incubated for 2 h in the dark with an Alexa fluor 555-conjugated anti-mouse IgG (Cell Signaling Technology, Danvers, MA, USA). The cells were visualized with an inverted fluorescence microscope (U-HGLGPS, OLYMPUS, Tokyo, Japan). In addition, the Vero cells were harvested at 24 h post virus infection and were subjected to Western blot analysis using gB protein antibody (1:2000) and goat anti-mouse IgG antibody (Solarbio, Shanghai, China).

### 2.2. Growth Curves

The isolated viruses were propagated for viral titer determination calculated as 50% tissue culture infectious dose per mL (TCID_50_/mL) using the Reed–Muench method. A monolayer of Vero cells was infected with the stocked virus at a multiplicity of infection (MOI) of 0.1 for 1 h. After washing, fresh DMEM containing 2% FBS was added. The supernatant and cells were harvested every 12 h post infection for titer determination through the observation of CPEs by 72 h post infection.

### 2.3. Identity, Genetic Variations, and Phylogenetic Analysis

The whole genome of the PRV variants was extracted according to a method published before [11]. Purified genomic DNA was sequenced through next-generation sequencing (NGS) technology using Illumina paired-end sequencing (Sangon Biotech, Shanghai, China), and the genome sequence was annotated using SnapGene 6.0 software. Twenty representative PRV reference strains (not all available sequences) were downloaded from the NCBI database for identity and phylogenetic analyses. Information on the referenced strains is shown in Table 1. The complete genomic sequence and deduced amino acids were aligned and compared using DNASTAR Lasergene. v7.1. Phylogenetic trees based on the complete genome and major immunogenic and virulence-related genes (gB, gC, gD, and gE) were constructed using MEGA 7.0 with the maximum likelihood (ML) method, the Kimura 2-parameter model, and 1000 bootstrap replication.

### 2.4. Animal Experiments of Three Isolates

A total of sixteen 8-week-old growing pigs without classical swine fever virus (CSFV), PRV, porcine circovirus 2 (PCV2), and porcine reproductive and respiratory syndrome virus (PRRSV) pathogen (negative using qRT-PCR test kits from Jiazhi, Qingdao, China) and PRV gB and gE antibody (negative using ELISA kits from IDEXX, Westbrook, ME, USA) were randomly assigned into four groups (four pigs in each group). After a 1-week acclimatization period, pigs in the challenge groups were each intranasally inoculated with SD1501, SD1701, or SD1801 (2 mL, 10^7^ TCID_50_/mL). Group 4 served as the unchallenged control group and was inoculated intranasally with 2 mL of DMEM. Rectal temperatures and clinical signs were recorded daily for 8 days following inoculation. Gross clinical symptoms, respiratory symptoms and neurological symptoms were scored according to the evaluation criteria listed in Supplementary Table S1. The average daily weight gain (ADWG) was calculated at 7 days post inoculation (dpi). The pigs developed severe clinical symptoms, such as muscle spasms, inability to stand, and dying respiratory difficulties during the experimental period, and all remaining pigs at 8 dpi were humanely euthanized (with an injection of pentobarbital sodium, 150 mg/kg). Post-mortem, lung, brain, and lymph node tissues were collected and fixed in 10% formaldehyde. Hematoxylin and eosin staining was applied for histopathological examination, and PRV gB specific antibody (1:200) was used for immunohistochemistry. The animal experiment was approved by the animal welfare and ethics committee of Yangzhou University (202409007), and all procedures were performed according to conventional animal welfare guidelines.

### 2.5. Statistical Analysis

The data were processed and analyzed using GraphPad Prism 8.0 software (GraphPad, San Diego, CA, USA).

## 3. Results

### 3.1. Isolation and Identification of SD1501, SD1701, and SD1801

**Figure 1:**
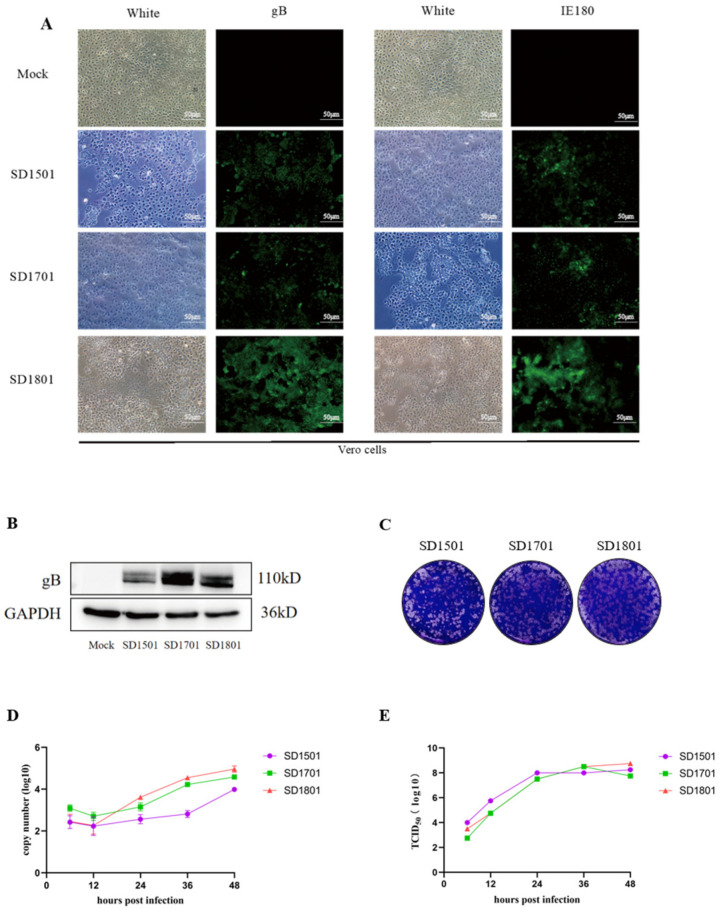
Isolation, identification, and biological characteristics of the three isolated field PRV strains. (**A**) Vero cells were inoculated with SD1501, SD1701, and SD1801, and the cytopathic effects and fluorescence were observed. (**B**) Identification of SD1501, SD1701, and SD1801 using Western blot analysis. (**C**) Plaque purification of SD1501, SD1701, and SD1801. (**D**,**E**) Growth curves of SD1501, SD1701, and SD1801 using qPCR (**D**) and TCID50 determination (**E**).

Three homogenized PRV positive samples reserved in 2015, 2017, and 2018 were inoculated to Vero cells for virus isolation. Through three successive blind passages, the cells showed typical PRV CPEs, characterized by cell rounding, fragmentation, and floating (Figure 1A). The indirect immunofluorescent assay (IFA) using the specific PRV gB and IE180 antibody demonstrated the presence of infectious PRVs (Figure 1A), with a Western blot confirmation of 110 kDa gB glycoprotein (Figure 1B). At 48 h post infection (hpi), the third-passage supernatants were collected and subjected to two rounds of plaque purification. As shown in Figure 1C, three PRV strains named SD1501, SD1701, and SD1801 were successfully isolated with similar plaque phenotypes. Moreover, growth curves were determined to analyze the biological characteristics of the isolated strain using qPCR and a TCID_50_ assay (Figure 1D,E), which showed that the three isolates grew well and there was no significant difference among them in Vero cells. The peaked viral titers of the SD1501, SD1701, and SD1801 isolates were 10^9.25^, 10^9.5^, and 10^9.75^ TCID_50_/mL, respectively.

### 3.2. Whole-Genome Sequencing

The genomes of SD1501, SD1701 and SD1801 isolates were extracted and sequenced through second-generation sequencing technology. Following assembly using the referenced PRV sequences, the complete genome length of SD1501, SD1701, and SD1801 was 141,255 bp, 143,098 bp, and 143,110 bp, respectively, with similar UL, US, IR, and TR parts consisting of 69 open reading frames (ORFs) and 74% GC content. Comparing the complete genome sequence with the other referenced strains, no structural rearrangements or gene deletions were observed. The linear map, gene arrangement, and distribution of the complete genome sequence were annotated using the Snapgene software (Figure S1). The sequence of SD1501, SD1701, and SD1801 were deposited in GenBank with the accession numbers PV157260, PV157262, and PV157261.

### 3.3. Identity Alignment and Phylogenetic Analysis

**Figure 2:**
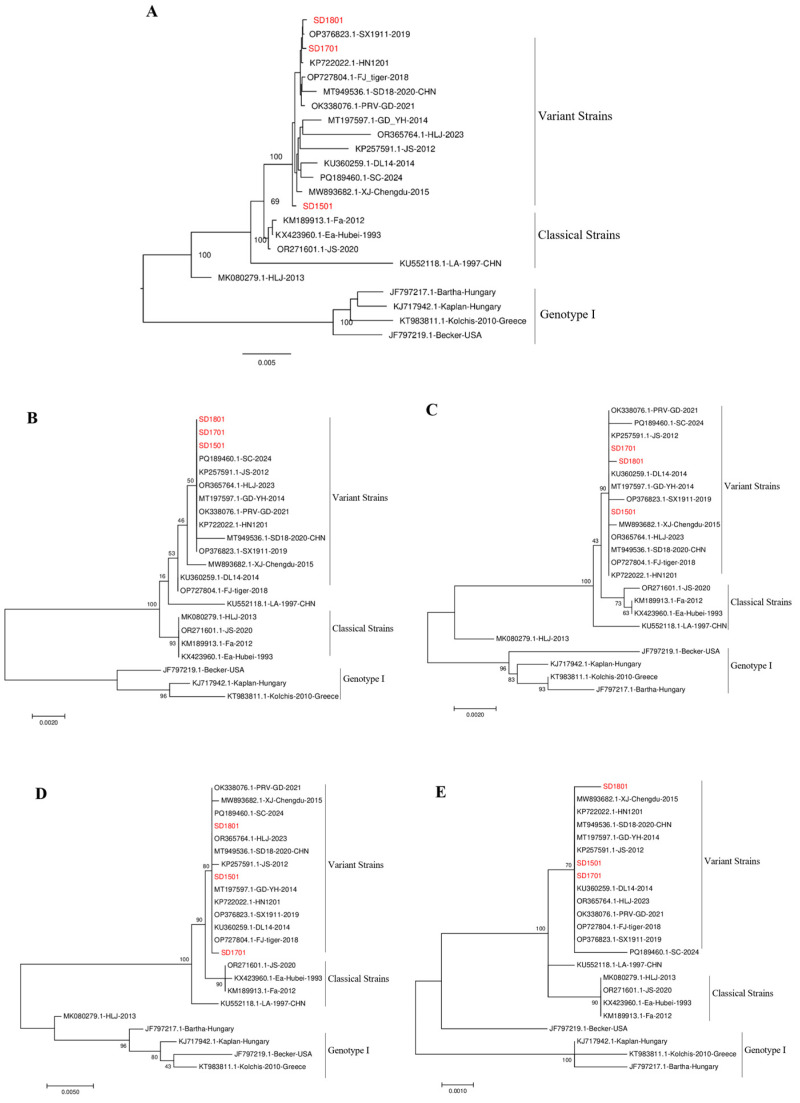
Phylogenetic analysis of the three isolated field PRV strains. The phylogenetic trees based on the complete genome (**A**), gB (**B**), gC (**C**), gD (**D**), and gE (**E**) were constructed using the MEGA 7.0 software with the maximum likelihood (ML) method, and all branch lengths were drawn to a scale of nucleotide substitutions per site. The isolated SD1501, SD1701, and SD1801 strains are indicated by red color.

Based on the identity comparisons of the complete genome sequence of SD1501, SD1701, and SD1801, it was shown that the three isolates shared 99.8%, 99.9%, and 99.9% similarity with Chinese variant strain HN1201, and 99.7%, 99.5%, and 99.6% identity with classical strain Ea, respectively. In contrast, identity with the Bartha strain was significantly lower, at 96.6%, 97.0%, and 97.0%, respectively (Table 2). As shown in Table 3, the identity of major virulent and immunogenic-related genes was also analyzed and exhibited similar results to the complete genome comparisons. In addition, characteristic molecular markers identical to genotype II strains were observed when performing the amino acid alignment of isolates with referenced strains, including amino acid deletion (^75^SPG^77^) in gB, insertion (^63^AAASTPA^69^) in gC, deletion (^280^RP^281^) in gD, and insertion (^497^D) in gE (Figure S2). Phylogenetic trees constructed using the maximum likelihood (ML) method, based on the complete genome and gE, gB, gC, and gD genes, further supported these findings. As shown in Figure 2, all three isolates clustered into the same branch as genotype II variant strains. Therefore, combining the identity and phylogenetic analyses, the isolates of SD1501, SD1701, and SD1801 were demonstrated to be Chinese variant strains of genotype II.

### 3.4. Amino Acid Variations in SD1501, SD1701, and SD1801 Compared to Variant Strain HN1201

Previous studies primarily focused on several immunogenic and virulence-related proteins. To have better knowledge of virus evolution, a comprehensive analysis of amino acid variations across 68 ORFs was conducted between the isolated strains and the representative variant strain HN1201. Compared to HN1201, most ORFs were highly conserved, and a total of 8, 8, and 13 proteins of the SD1501, SD1701, and SD1801 strains were different, including mutations, deletions, and insertions (Supplementary Tables S2–S4). Among the variant proteins, four proteins (UL24, UL12, gG, and US9) were common to SD1501, SD1701, and SD1801. The same amino acid variations were identified as shared between the three isolates, namely, UL24 (^G^85^R^), UL12 (^V^2^A^), gG (^S^222^G^), and US9 (^T^79^A^). Notably, UL24 is an important tegument protein critical for immune response evasion, and gG is a glycoprotein essential for cell-to-cell spread and virulence. The potential impact of these variations on pathogenicity warrants further investigation. In addition to shared variations, strain-specific differences were observed. SD1501 exhibited a unique deletion of nine amino acids in UL5 (a key protein of viral genome DNA synthesis), SD1701 displayed a unique mutation and deletion in IE180, while SD1801 showed specific mutations in gB, gD, and gI (major immunogenic and virulence-related proteins) (Supplementary Tables S2–S4). These findings suggest a potential correlation between viral genome variability and pathogenicity, providing valuable insights for future genotype–phenotype studies.

### 3.5. Pathogenicity Analysis of SD1501, SD1701, and SD1801

**Figure 3:**
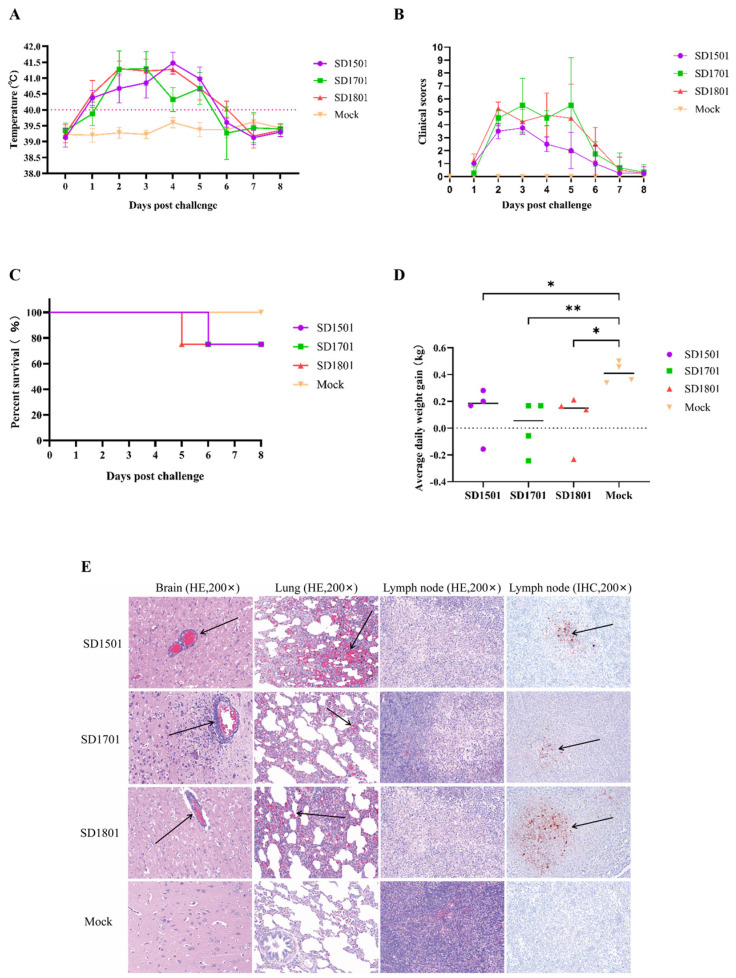
Pathogenicity analyses of SD1501, SD1701, and SD1801 in pigs. The daily temperature (**A**), scores of clinical signs (**B**), survival percentage (**C**), and average daily weight gain (**D**) were analyzed. (**E**) Histopathological examination (HE) and immunohistochemistry staining (IHC) results of the brain, lungs, and lymph nodes infected with the three isolated strains. Original magnification 200×. * and ** indicated a statistically significant difference (*p* < 0.05 or *p* < 0.01). The arrows indicated the speficic histopathological changes and positive IHC stainings.

To evaluate pathogenicity, 9-week-old pigs in each group were challenged intranasally with 2 mL (10^7^ TCID_50_/mL) of SD1501, SD1701, or SD1801. The results showed that all infected pigs developed a fever exceeding 40 °C within 2 dpi (Figure 3A). Notably, three pigs in the SD1701 group and four pigs in the SD1801 group exhibited high fever (>41 °C). By 4 dpi, all four infected pigs in the SD1501 and SD1801 groups displayed rectal temperatures above 41 °C, which persisted above 40 °C until 6 dpi. From 1 dpi, clinical symptoms (depression, aggregation, and reduced feed and water intake) were observed in the challenged groups, and the pigs showed typical PR symptoms by 2 dpi, such as respiratory distress, salivation, convulsion, and paddling. Severe outcomes were observed in some pigs: one SD1801-infected pig died at 5 dpi due to severe dyspnea, one SD1701-infected pig was unable to stand and succumbed to muscle tremors at 6 dpi, and one recumbent pig with paddling in the SD1501 group died at 6 dpi. As shown in Figure 3B, the clinical score returned to normal after 5 dpi. No aberrant signs were observed in the DMEM-treated control group. In addition, compared to the control group, all the infected pigs exhibited lower average daily weight gain (ADWG), and no significant differences were observed among the three groups (Figure 3D). In a previous study, Yang et al. reported that the variant strain HN1201 exhibited 100% mortality in 60- and 127-day-old pigs within 8 dpi [18]. In contrast, the lower mortality observed in this study suggests that SD1501, SD1701, and SD1801 may exhibit reduced pathogenicity compared to HN1201.

All the surviving and moribund pigs were euthanized for gross and microscopic pathological examinations at 8 dpi. After autopsy, no obvious gross lesions were observed in most of the challenged pigs. However, the moribund pigs exhibited edema and hemorrhage in the brain and consolidation in lung tissues. The brain, lung, and lymph nodes were collected for histological examination (HE) and immunohistochemistry staining (IHC). As shown in Figure 3E, nonsuppurative encephalitis characterized by lymphocyte infiltration around the small blood vessels, hemorrhage and alveolar interstitial thickening in the lungs, and necrosis in lymph nodes were observed in PRV infected pigs. In accordance with the HE results, the IHC staining of lymph node samples showed strong positive signals in the infected groups compared to the mock group (Figure 3E).

## 4. Discussion

PRV has emerged as a significant concern for the swine industry worldwide, particularly in China, with the emergence of variant strains since late 2011. Although PRV modified live vaccines (MLVs) have greatly contributed to the control of PR in the field, some cases or outbreaks have been closely linked to the emergence of recombinant strains derived from MLV viruses in recent years, especially from classical vaccines, which may be due to their wide and massive use in swine herds [21]. It is well known that the severity of clinical symptoms associated with PRV infection is inversely correlated with the age of the infected pigs [22]. However, Yang et al. found that the variant strain HN1201 exhibited 100% mortality in 35-, 60-, and 127-day-old pigs following intranasal inoculation with 10^7^ TCID_50_, indicating unexpected age-independent high virulence [18]. Following the outbreak, PRV variants with increased pathogenicity rapidly compromised most pig farms, prompting numerous studies to focus on the epidemiological and genetic characteristics [23,24]. While many of these studies evaluated pathogenicity in mice or rats, limited data are available on the pathogenicity of PRV field variants in pigs, particularly for strains circulating between 2015 and 2018. For instance, two-month-old piglets infected with PRV-GD and PRV-JM (10^6^ TCID_50_), isolated in 2022, succumbed to infection 6 dpi [25]. In 2024, Xu et al. analyzed the pathogenicity of four genotype II variant strains (MS, XJ, LS, and CZ) using two-week-old piglets, reporting that infected piglets began to die by 3 dpi, with all piglets succumbing within 8 days [26]. Another study published in 2024 reported that the intranasal inoculation of suckling piglets with 2 mL of variant TJbd2023 strain (10^6.57^/0.1 mL) resulted in a mortality rate of 66.70% [27]. In the present study, three PRV strains named SD1501, SD1701, and SD1801 were isolated from 2015 to 2018, and were all identified as genotype II Chinese variant strains based on identity and phylogenetic analyses.

To evaluate their pathogenicity, 9-week-old growing pigs were intranasally inoculated with each isolate. The results revealed typical transient PRV clinical signs, with no obvious gross lesions observed in most challenged pigs upon autopsy. The mortality rate across all three challenge groups was 25%. Compared to the highly pathogenic HN1201 (the representative earliest variant strain) and the limited pathogenicity data available for other variants in pigs, SD1501, SD1701, and SD1801 exhibited lower pathogenicity in growing pigs. As is well known, to balance efficient replication and transmission with the risk of host mortality, achieving virus evolution toward intermediate virulence is one of the most important directions. This trend has been observed in several swine disease viruses, such as African swine fever virus (ASFV), classical swine fever virus (CSFV), porcine reproductive and respiratory syndrome virus (PRRSV), and porcine epidemic diarrhea virus (PEDV) [28,29,30,31]. Therefore, we speculate that the emergence of moderately pathogenic PRV strains three to four years after the initial appearance of highly pathogenic variants in 2011 represents a plausible evolutionary trajectory. In the field, for growing pigs, particularly those aged 10 to 13 weeks, milder clinical signs (such as transient fever, reduced appetite, and mild respiratory distress without high mortality) are frequently observed and may be associated with the emergence of moderately pathogenic PRV strains. To better mimic natural infection, moderate strains may serve as ideal challenge viruses for evaluating novel vaccines. Contrary to what might be assumed, i.e., that the moderate PRV variants may be beneficial for clinical prevention and control, their reduced pathogenicity could be of particular concern. The absence of dramatic clinical manifestations complicates disease diagnosis and surveillance, potentially leading to misdiagnosis and persistent infections due to the similar clinical presentation of PRRSV, PCV2 cases, and other management factors. Furthermore, long-term or latent infections in swine populations could serve as a reservoir for virus evolution and recombination and the emergence of novel strains [32,33]. Therefore, continued surveillance, improved diagnostic methods, and more precise risk assessments and attention will be essential to mitigate the impact of variants with moderate pathogenicity. In particular, extended holding and quarantine time should be applied to confirm asymptomatic strains when introducing gilts.

The evolution of PRV is a complex and multifaceted process driven by a series of factors, including mutation, natural selection, recombination, host–virus interaction, environmental conditions, and anthropogenic pressures. It was reported that neurotropism and the induction of immune response are the key determinants of the pathogenicity of PRV, which was highly associated with immunogenic and virulence-related genes [34,35]. To understand the mechanism potentially underlining reduced virulence, the amino acids variations in gB, gC, gD, gE, gI, and TK were firstly analyzed. Compared to the earlier highly pathogenic HN1201 strain, only SD1801 showed specific mutations in the gB, gD, and gI proteins. Additionally, four proteins with the same amino acid variations, namely, UL24 (^G^85^R^), UL12 (^V^2^A^), gG (^S^222^G^), and US9 (^T^79^A^), were identified in SD1501, SD1701, and SD1801 based on the comparison of the 68 ORFs. Further investigation into the genetic determinants of moderate pathogenicity and longitudinal epidemiological studies are needed, which would provide critical data for understanding the evolution of PRV and modifying control strategies in the field. Notably, we only focused on the characterization and pathogenicity analyses of the three isolated variants and could not determine the precise difference in the onset, course, viral load, and pathogenic results compared to the highly pathogenic strains, which was an unexpected limitation in the present study. Further research should address these limitations by performing animal experiments with diverse strains at the same time and carrying out comprehensive analyses from neuropathogenesis and the induction of cytokines.

## 5. Conclusions

In summary, three PRV field strains, SD1501, SD1701, and SD1801, were isolated and characterized as genotype II Chinese variant isolates based on identity and phylogenetic analyses. All three isolates exhibited moderate pathogenicity, with a mortality rate of 25% in 9-week-old growing pigs. Our results provide evidence of different phenotypes caused by PRV infection and contribute to a better understanding of PRV evolution in China.

## Figures and Tables

**Table 1:** Information of referenced pseudorabies virus strains obtained from GenBank.

Strain	Accession Number	Country	Isolation Date
SX1911	OP376823.1	China	2019
HN1201	KP722022.1	China	2016
FJ_tiger	OP727804.1	China	2018
SD18-2020	MT949536.1	China	2020
PRV-GD	OK338076.1	China	2021
GD_YH-2014	MT197597.1	China	2014
HLJ-2023	OR365764.1	China	2023
JS-2012	KP257591.1	China	2012
DL14-2014	KU360259.1	China	2014
SC-2024	PQ189460.1	China	2024
PRV XJ	MW893682.1	China	2015
Fa	KM189913.1	China	2012
Ea-Hubei	KX423960.1	China	1993
JS-2020	OR271601.1	China	2020
HLJ-2013	MK080279.1	China	2013
Bartha	JF797217.1	Hungary	1961
Kaplan	KJ717942.1	Hungary	2014
Kolchis-2010	KT983811.1	Greece	2010
Becker	JF797219.1	USA	1970
LA	KU552118.1	China	1997

**Table 2:** Nucleotide identities of SD1501, SD1701, and SD1801 compared to referenced strains.

Strain	Nucleotide Identities (%)					
	Classical Strain		Variant Strain		Genotype I	
	Ea	Fa	HN1201	JS-2012	Bartha	Becker
SD1501	99.7	99.4	99.8	99.4	96.6	96.7
SD1701	99.5	99.6	99.9	99.3	97.0	97.1
SD1801	99.6	99.6	99.9	99.3	97.0	97.1

**Table 3:** Amino acid identities of major proteins compared to referenced strains.

Strain	Protein	Amino Acid Identities (%)					
		Classical Strain		Variant Strain		Genotype I	
		Ea	Fa	HN1201	JS-2012	Bartha	Becker
SD1501	gB	99.5	99.5	100.0	100.0	96.9	97.5
	gC	99.2	99.4	100.0	99.8	93.1	93.3
	gD	99.6	99.6	100.0	100.0	98.9	99.3
	gE	83.3	99.1	100.0	100.0	\	95.8
	gI	100.0	100.0	100.0	100.0	100.0	94.5
	TK	100.0	100.0	100.0	100.0	99.4	99.4
SD1701	gB	99.5	99.5	100.0	100.0	96.9	97.5
	gC	99.0	99.2	99.8	99.6	92.9	93.1
	gD	99.6	99.6	100.0	100.0	98.9	99.3
	gE	83.3	99.1	100.0	100.0	\	95.8
	gI	100.0	100.0	100.0	100.0	100.0	94.5
	TK	100.0	100.0	100.0	100.0	99.4	99.4
SD1801	gB	99.3	99.3	99.9	99.9	96.8	97.4
	gC	99.2	99.4	100.0	99.8	93.1	93.3
	gD	99.5	99.5	99.9	99.9	98.8	99.2
	gE	83.3	99.1	100.0	100.0	\	95.8
	gI	99.7	99.7	99.7	99.7	100.0	94.3
	TK	100.0	100.0	100.0	100.0	99.4	99.4

## Data Availability

The original contributions presented in the study are included in the article/Supplementary Material, and further inquiries can be directed to the corresponding authors.

## Supplementary Materials

The following supporting information can be downloaded at: https://www.mdpi.com/article/10.3390/microorganisms13040851/s1, Figure S1: Diagrammatic genome distribution of isolated strains; Figure S2: Amino acid alignment of gB, gC, gD, and gE proteins. Table S1: The symptom and scoring criteria used in this study; Table S2: Variant proteins and amino acids of SD1501 compared to HN1201 strain; Table S3: Variant proteins and amino acids of SD1701 compared to HN1201 strain; Table S4: Variant proteins and amino acids of SD1801 compared to HN1201 strain.

## Abbreviations

The following abbreviations are used in this manuscript:

PRV	Pseudorabies virus
TCID_50_	50% tissue culture infective dose
DMEM	Dulbecco’s modified Eagle medium
PBS	Phosphate-buffered saline
CPE	Cytopathic effects
IFA	Immunofluorescent assay
CSFV	Classical swine fever virus
PRRSV	Porcine reproductive and respiratory syndrome virus
ADWG	Average daily weight gain
ORF	Open reading frame
ASFV	African swine fever virus
PEDV	Porcine epidemic diarrhea virus
