# Supplementary material for: Emergence and Characterization of Three Pseudorabies Variants with Moderate Pathogenicity in Growing Pigs
Source: Microorganisms. 2025 Apr 9;13(4):851. doi: 10.3390/microorganisms13040851 (PMC12029256; doi:10.3390/microorganisms13040851)
Supplement: Supplementary file 1 [file microorganisms-13-00851-s001.zip › microorganisms-3537298-supplementary.pdf]

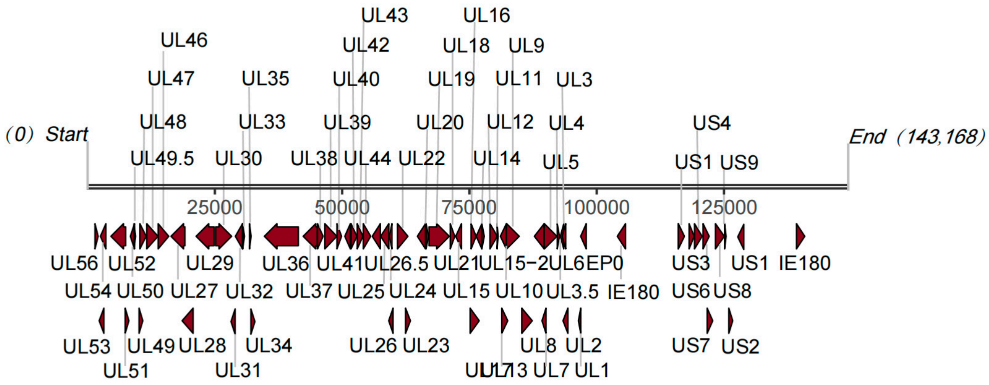

Figure S1. The diagrammatic genome distribution of the isolated strain.

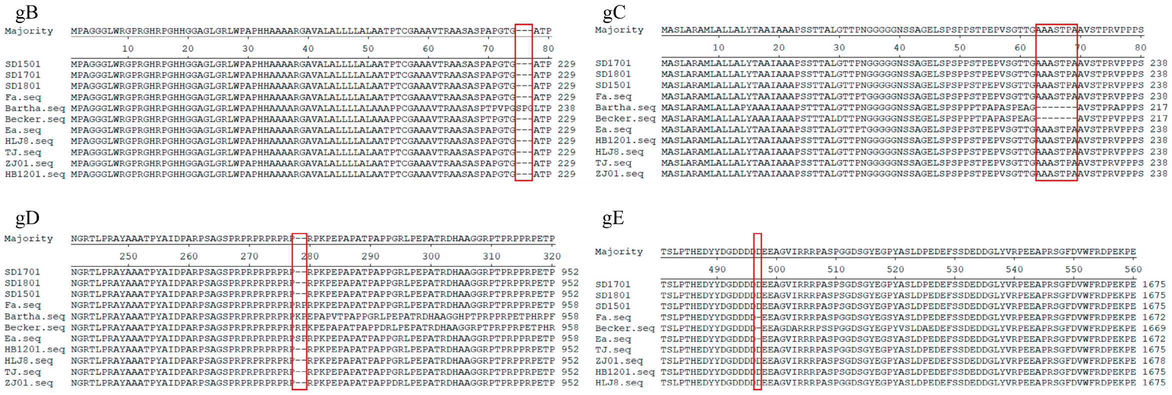

Figure S2. Amino acids alignment of the gB, gC, gD and gE proteins. The deletions and insertions were marked with the red box.

Table S1. The symptom and scoring criteria used in the study.

Symptom type		Scoring criteria	Score
Gross clinical symptom	Temperature	T≤39.9℃	0
		40.0℃≤T≤40.9℃	1
		41.0≤T	2
	Appetite	Normal	0
		Loss of appetite	1
	Mentality	Normal	0
		Depressed / Lethargic	1
Respiratory symptom	Respiratory symptom	Normal	0
		Asthma	1
		Shortness of breath at rest	2
		Shortness of breath and dyspne a	3
		Laborious	4
	Cough	Normal	0
		Cough	1
	Runny nose	Normal	0
		Runny nose	1
Neurological symptom	Neurological symptom	Normal	0
		Shivering	1
		Ataxia	2
		Four limb stroking	3
		Paralysis	4

Table S2. The variant proteins and amino acids shared of SD1501 compared to HN1201 strain.

Protein Name	Number (aa)	Amino Acid Residues and Location
UL46	1	R435H
UL37	1	D844G
UL24	1	G85R
UL12	1	V2A
UL5	9	570-578(ΔAGPGGPGGP)
gG	1	S222G
US9	1	T79A
IE180	6	862-867(+STKSSS)

Table S3. The variant proteins and amino acids shared of SD1701 compared to HN1201 strain.

Protein Name	Number (aa)	Amino Acid Residues and Location
UL37	1	A156V
UL24	1	G85R
UL17	1	D176Y
UL12	1	V2A
UL7	1	P253T
gG	1	S222G
US9	1	T79A
IE180	4	A177T 441(+S) 840(ΔA) S1366G

Table S4. The variant proteins and amino acids shared of SD1801 compared to HN1201 strain.

Protein Name	Number (aa)	Amino Acid Residues and Location
UL46	1	563(ΔE)
gB	1	H242R
UL30	1	A2V
UL37	1	E10A
UL24	1	G85R
UL20	1	A54V
UL17	1	L445P
UL12	1	V2A
gG	2	S222G D253N
gD	1	L366R
gI	1	T156M
US9	2	L22P T79A
IE180	2	441(+S) P1441L
